# A Model Incorporating Serum Alkaline Phosphatase for Prediction of Liver Fibrosis in Adults with Obesity and Nonalcoholic Fatty Liver Disease

**DOI:** 10.3390/jcm10153311

**Published:** 2021-07-27

**Authors:** Ahmad Hassan Ali, Gregory F. Petroski, Alberto A. Diaz-Arias, Alhareth Al Juboori, Andrew A. Wheeler, Rama R. Ganga, James B. Pitt, Nicole M. Spencer, Ghassan M. Hammoud, R. Scott Rector, Elizabeth J. Parks, Jamal A. Ibdah

**Affiliations:** 1 Division of Gastroenterology and Hepatology, University of Missouri, Columbia, MO 65212, USA; aliah@health.missouri.edu (A.H.A.); aljubooria@health.missouri.edu (A.A.J.); hammoudg@health.missouri.edu (G.M.H.); rectors@health.missouri.edu (R.S.R.); parksej@missouri.edu (E.J.P.); 2 Biostatistics and Research Design Unit, School of Medicine, University of Missouri, Columbia, MO 65212, USA; PetroskiG@health.missouri.edu; 3 Boyce & Bynum Pathology Professional Services, Columbia, MO 65201, USA; ADiazArias@bbpllab.com; 4 Department of Surgery, University of Missouri, Columbia, MO 65212, USA; wheeleraa@health.missouri.edu (A.A.W.); gangar@health.missouri.edu (R.R.G.); jpitt@columbiasurgical.com (J.B.P.); spencernm@columbiasurgical.com (N.M.S.); 5 Research Service, Harry S Truman Memorial Veterans Medical Center, Columbia, MO 65201, USA; 6 Department of Nutrition and Exercise Physiology, School of Medicine, University of Missouri, Columbia, MO 65212, USA; 7 Department of Medical Pharmacology and Physiology, School of Medicine, University of Missouri Columbia, Columbia, MO 65212, USA

**Keywords:** nonalcoholic fatty liver disease, metabolic surgery, liver fibrosis, histology, alkaline phosphatase

## Abstract

We assessed the relationship between serum alkaline phosphatase (ALP) and liver fibrosis by histology, in addition to other noninvasive parameters, in obese patients undergoing metabolic surgery. Patients scheduled for elective bariatric surgery were prospectively recruited from a bariatric clinic. An intraoperative liver biopsy was performed, and liver histology was evaluated by a pathologist blinded to the patients’ data. The endpoint was significant fibrosis defined as fibrosis stage ≥ 2. Independent predictors of fibrosis were identified by logistic regression. Two hundred ten patients were recruited. Liver histology revealed steatosis in 87.1%, steatohepatitis in 21.9%, and significant fibrosis in 10%. Independent predictors of significant fibrosis were ALP (Odds Ratio (OR) 1.03; 95% Confidence interval (CI), 1.01–1.05), alanine aminotransferase (OR 1.02; 95% CI, 1.01–1.03), HbA1c (OR 1.58; 95% CI, 1.20–2.09), and body mass index (OR 1.06; 95% CI, 1.00–1.13). A tree-based model was developed to predict significant fibrosis, with a receiver operating characteristic (ROC) area of 0.845, sensitivity of 0.857, specificity of 0.836, and accuracy of 0.931. The applicability of serum ALP as an independent biomarker of liver fibrosis should be considered in obesity surgery patients, and in the broader context of obese patients with nonalcoholic fatty liver disease.

## 1. Introduction

The term nonalcoholic fatty liver disease (NAFLD) encompasses a wide spectrum of liver diseases characterized by fatty infiltration of the liver. When inflammation is present, the term nonalcoholic steatohepatitis (NASH) is used. NAFLD can be complicated by fibrosis, an important predictor of outcomes in patients with NAFLD [1,2,3,4]. The increased incidence of NAFLD has paralleled a continued rise in the prevalence of morbid obesity. Metabolic surgery has been reported to induce weight loss, improve type 2 diabetes, induce resolution of NASH, and lead to regression of fibrosis [5,6,7,8,9].

A tremendous interest exists in utilizing noninvasive, readily available, and inexpensive clinical parameters as means of assessing the stage of fibrosis in patients with chronic liver disease. Several models have been developed to serve that purpose [10,11,12]. However, these models have been developed largely from patients diagnosed with NASH to predict advanced fibrosis (F3–F4); therefore, their applicability in obese patients undergoing elective metabolic surgery is limited due to the low prevalence of moderate-to-severe stage NASH in this population [13]. Thus, there is a need for studies that address predictors of liver fibrosis in this patient population.

Biomarkers of cholestasis, namely serum alkaline phosphatase (ALP), have been widely used as prognostication tools in cholestatic liver diseases [14]. However, the significance of serum ALP in NAFLD, particularly in obese patients undergoing metabolic surgery, has been rarely addressed. Other abnormalities associated with obesity, including the presence of hypertension, insulin resistance, type 2 diabetes, sleep apnea, and liver enzymes abnormalities have been used to predict NASH in patients undergoing metabolic surgery [15]. In a small cohort of patients with liver disease, 10% of patients with NAFLD/NASH presented with isolated serum ALP elevation with normal transaminases levels, of whom nearly one-third were found to have advanced liver fibrosis at the time of presentation [16]. Thus, serum ALP may be of prognostic relevance in patients with NAFLD and its role as a predictor of liver fibrosis, particularly in obese patients undergoing metabolic surgery, requires further investigation. Given the rapidly growing burden of NAFLD and obesity, assessment of fibrosis risk in obese patients undergoing metabolic surgery is instrumental in the management of these patients.

The goals of this prospective study were to: (a) identify clinical and laboratory predictors of significant fibrosis in this patient population, (b) assess the relationship between serum ALP levels and fibrosis stage by liver histology, and (c) develop a model to predict significant fibrosis in obese patients with NAFLD.

## 2. Materials and Methods

### 2.1. Study Participants

Liver samples were obtained from adults with clinical obesity undergoing elective metabolic surgery at the University of Missouri Hospital, Columbia (MO, USA). Before inclusion, all participants gave written informed consent to the protocol, which was approved by the institutional review board (IRB) of University of Missouri (protocol #2008258) and conducted according to World’s Medical Association 1964 Declaration of Helsinki and its later amendments. This study is registered at ClinicalTrials.gov (Identifier: NCT03151798). In preparation for metabolic surgery, a standardized duration of educational instruction including a high-protein, liquid diet that was low in carbohydrate prescribed 1–2 weeks before surgery [17]. Fasting blood samples were collected before anesthesia for measurement of the fasting lipid profile, complete blood count, blood glucose, hemoglobin A1c (HbA1c), alkaline phosphatase (ALP), aspartate aminotransferase (AST), alanine aminotransferase (ALT), and albumin at a CLIA-certified laboratory. To minimize the potential risk of liver injury and the resultant influx of inflammatory cells caused by anesthesia and liver manipulation [18,19], liver tissue was obtained after initiation of anesthesia according to standardized protocols via a wedge biopsy [8,20,21]. Hematoxylin and Eosin stain, Masson’s trichrome stain, and iron stain were performed. All specimens were interpreted, graded, and staged by a single experienced liver pathologist, who was blinded to the clinical and laboratory data of the study subjects. Grading for NASH and staging for fibrosis were performed utilizing the NASH Clinical Research Network NAFLD activity score (NAS) and fibrosis scoring system, respectively [22]. NASH was defined as NAS ≥ 5. Significant fibrosis was defined as fibrosis stage ≥ 2, whereas advanced fibrosis was defined as fibrosis ≥ 3. NAS inclusion criteria for NAFLD patients were based on an alcohol intake lesser than 20 g/day and histologically confirmed steatosis with/without necro-inflammation and/or fibrosis. Other causes of liver disease were excluded based on history, laboratory data, and histological features.

### 2.2. Statistical Analysis

Continuous variables were expressed as median with range, and categorical data were expressed as frequency and percentage. Study subjects were grouped according to the presence or absence of significant fibrosis (F0–F1 vs. F2–F4). The main endpoint of the study was to identify potential predictors of significant fibrosis (fibrosis stages 2–4) from routine blood panels. Fibrosis stage ≥ 2 was chosen as the definition for significant fibrosis because a recent large meta-analysis showed that the risk of liver-related death in NAFLD patients increases significantly after progression of fibrosis to stage 2 or higher [23]. Histograms were used to assess the normality of the continuous variables. Baseline clinical and laboratory variables (Table 1) were compared between the two groups using Chi-squared test for nominal scale variables and the Mann-Whitney test for interval scale variables.

#### 2.2.1. Identifying Risk Factors for Significant Fibrosis

To identify a minimal and reliable set of prognostic factors, each of the characteristics in Table 1 was regarded as a candidate predictor in a logistic regression analysis. A single iteration of a variable selection algorithm maximizes model fit to the sample data but in doing so the selected model may be strongly dependent on sample idiosyncrasies rather than a reflection of population characteristics. Variable selection was by backwards elimination using the minimum Akaike Information Criterion as the stopping rule [24]. To minimize selection artifacts variable selection was performed on each of 1000 bootstrap samples [25]. Variable importance was determined by the number of times each variable remained in the selected model and by examining the pairwise selection frequencies [24]. Variables selected in at least 50% of the iterations and that occurred with high frequency in the pairwise analysis were deemed likely to be important and not representing a false discovery. A final simple risk model is proposed. The final parameter estimates, odds ratios, and the area under ROC curves are estimated from second set of 1000 bootstrap replicates.

#### 2.2.2. Developing a Model for Predicting Significant Fibrosis

The identification of a minimal set of predictors for significant fibrosis is an essential first step. To make the findings immediately and easily useful to clinical practice we used tree-based methods in combination with a data augmentation technique to derive an algorithm for identifying patients at high risk for significant fibrosis. Classification trees are statistical models in which each predictor is partitioned for optimal classification of subjects with respect to a categorical outcome. A difficulty with trees is that they may not perform well when the prevalence of the outcome categories is extremely unbalanced. The problem is not unique to trees, rare outcomes are a problem for all classification techniques. The Synthetic Minority Over-sampling TEchnique (SMOTE) is an approach to the scarcity problem [26]. The fundamental SMOTE idea is to augment the original data with simulated representatives of the minority class (F2–F4), and then train a classifier on the augmented data set in which outcome prevalence is more nearly balanced. SMOTE starts by finding, in multidimensional space, the nearest neighbors to each member of the minority class and then adding a small random jitter to each of the predictors. The classifier is trained on the augmented data and then the original data or a validation data set, if available, is classified to evaluate the predictions.

Using the variables identified in the logistic regression analysis we applied SMOTE with matching on the five nearest neighbors for each member of the majority class. The final classification tree was grown and pruned under the following settings: (a) no variable was allowed to split more than once per path, (b) minimum allowable leaf size of five, (c) entropy was the measure of purity, and (d) cost-complexity pruning [27]. Statistical analyses were conducted using STATA version 12.1 (StataCrop LP, College Station, TX, USA). The implementation of SMOTE was based on code from Boardman et al. [28] and tree fitting was done with SAS/Stat HPSPLIT procedure.

## 3. Results

### 3.1. Subjects Characteristics

A total of 210 enrolled subjects had complete laboratory and histological data available for analysis. As shown in Table 1, the average age was 47 years, average body mass index (BMI) was 48 kg/m^2^, and 83% were female. Histologically, the prevalence of NAFLD and NASH were 87.1% (183/210) and 21.9% (46/210), respectively. Further, the prevalence of significant fibrosis (F2–F4) was 10% (21/210), while that of advanced fibrosis (F3–F4) was only 6.2% (13/210). Additional clinical, laboratory, and histological features are outlined in Table 1 and Table 2.

### 3.2. Risk Factors for Significant Fibrosis

Table 1 outlines the differences between those with significant fibrosis (F2–F4) vs. those with no or nonsignificant fibrosis (F0–F1). The frequency of patients with type 2 diabetes was significantly higher in those with significant fibrosis compared to those with no or nonsignificant fibrosis (67% vs. 29%; *p* < 0.0001). Furthermore, patients with significant fibrosis had significantly higher levels of serum glucose, HbA1c, ALP, AST, ALT, hemoglobin, and triglycerides compared to those with no or nonsignificant fibrosis (Table 1).

### 3.3. Predictors of Significant Fibrosis

**Figure 1:**
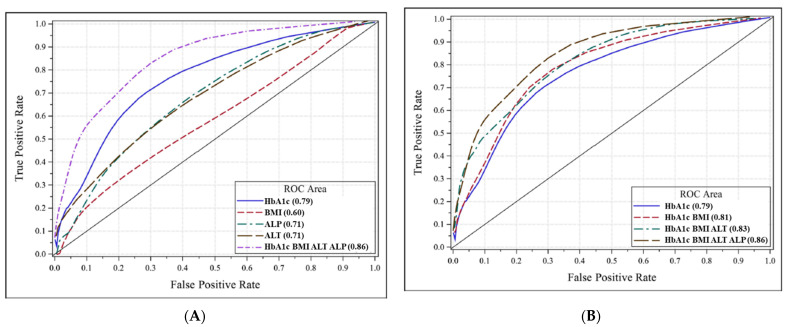
(**A**) The ROC curves for the individual variables HbA1c, BMI, ALT, and ALP, and for the proposed model combining the four variables for classification of patients undergoing metabolic surgery according to their fibrosis status (significant fibrosis (F ≥ 2) vs. no or nonsignificant fibrosis (F0–F1)). Abbreviations: ALT, alanine aminotransferase; ALP, alkaline phosphatase; BMI, body mass index; ROC, receiver operating characteristic. (**B**) The ROC curves for progressively larger models are displayed, illustrating the value of adding ALP to HbA1c, BMI, and ALT. All ROC areas are cross-validation estimates (data from *n* = 210). Abbreviations: ALT, alanine aminotransferase; ALP, alkaline phosphatase; BMI, body mass index; ROC, receiver operating characteristic.

Over the 1000 bootstrap sample analyses, 19 of the variables in Table 1 were selected with some frequency. Those variables that met the 50% iterations criteria were, in descending order of frequency, HbA1c, ALT, BMI, LDL/HDL ratio, ALP, hematocrit, and albumin. In the examination of pairwise frequencies only, HbA1c, ALT, BMI, LDL/HDL ratio, and ALP occurred in pairs with greater than 50% frequency. It was decided to exclude the LDL/HDL ratio from the final model as it represented a protective effect rather than a risk factor. Parameter estimates and odds ratios for the retained variables are given in Table 3. For the four-variable model presented in Table 3, the raw ROC area was 0.853 with a 95% confidence interval for area being 0.78 to 0.93. Figure 1A shows the ROC curve for the four-variable model compared to the ROC curve for each of the variables, whereas Figure 1B shows the ROC curves for progressively larger models with addition of each variable to illustrate the value of adding ALP to HbA1c, BMI, and ALT.

### 3.4. Model for Predicting Significant Fibrosis

**Figure 2:**
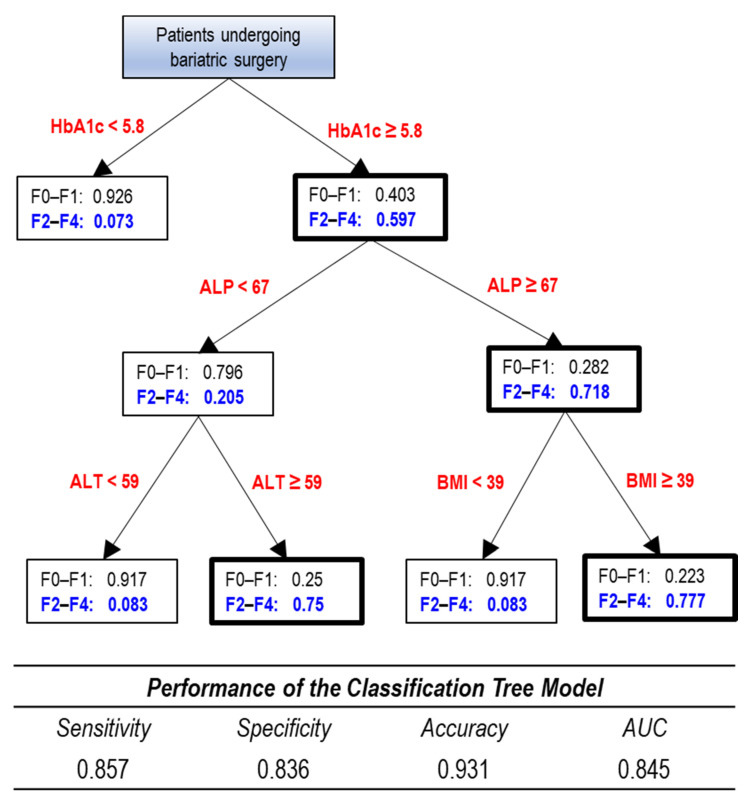
Classification tree model for discriminating between no fibrosis/nonsignificant fibrosis (F0–F1) vs. significant fibrosis (F2–F4) in obese patients undergoing bariatric surgery (*n* = 210). Numbers in the boxes represent probabilities. Significant fibrosis (F2–F4) group is typed in blue. Boxes dominated by the F2–F4 group are bolded. Values for HbA1c, ALP, BMI, and ALT are in %, U/L, kg/m^2^, and U/L, respectively. Abbreviations: ALP, alkaline phosphatase; ALT, alanine aminotransferase; AUC, area under curve; BMI, body mass index; HbA1c, hemoglobin A1c.

Using k = 5 as the number of nearest neighbors to each of the patients in the F2–F4 group (*n* = 21), the sample size of the F2–F4 group in the new SMOTE data set increased to 105. Thus, the new SMOTE data set consisted of the original F0–F1 cases (*n* = 189) plus the original F2–F4 cases (*n* = 21) plus the synthetic F2–F4 sample (*n* = 105), totaling 315 cases. Six synthetic cases were removed due to extreme distance in the nearest neighbor matching. Hence, the final SMOTE data set consisted of a total of 309 cases. The final predictive model with cutoff points for HbA1c, BMI, ALP, and ALT is presented as a decision tree (Figure 2). For the four-variable tree model, the raw ROC area was 0.845, with a sensitivity, specificity, and accuracy of 0.857, 0.836 and 0.931, respectively.

## 4. Discussion

In this prospective study of obese patients with NAFLD who were consecutively enrolled and underwent elective metabolic surgery with intraoperative liver tissue sampling, we report for the first time the prognostic relevance of serum ALP in these patients. Specifically, the present analysis identified that serum ALP levels can be useful in predicting liver fibrosis stage ≥2 in obese patients with NAFLD. Upon review of the literature, few bariatric studies were found that aimed to identify clinico-laboratory and histological predictors of fibrosis. Of the previously published nine studies that are relevant to the current study, serum ALP was not considered in the univariate/multivariate analysis [13,29,30,31,32,33,34,35,36]. It appears that the perception that serum ALP may not be as important as other known variables in predicting liver fibrosis in NASH may have led to excluding ALP from the analysis in many NALFD/NASH studies, particularly in obese patients with NAFLD undergoing elective metabolic surgery.

In this study, we developed a new tree-based model, incorporating serum ALP in addition to HbA1c, ALT, and BMI variables for the prediction of significant fibrosis (F2–F4) in our cohort. A recent large meta-analysis study showed that the risk of liver-related death in NAFLD patients increases significantly after progression of fibrosis to stage 2 or higher [23]. This model, shown in Figure 2, provides an algorithm based on noninvasive markers to identify probability for significant fibrosis in obese patients with NAFLD, which can be useful in deciding whether intraoperative liver biopsy is warranted in patients undergoing elective metabolic surgery. This model asserts that HbA1c is the primary marker for determining the probability for F2–F4 followed by serum ALP. It should be noted that the cutoff value for serum ALP for the purpose of assessing risk of liver fibrosis in this patient population falls within the normal range of serum ALP based on reference values (reference value for ALP in our laboratory is 33–130 U/L). However, it is important to recognize that reference values are different from clinical decision limits; while the reference values are based on test results in the normal population, the clinical decision limits are based on test results distribution in both the normal and diseased population in a particular disorder to assess risk for that disorder [37]. This is in line with the recommendations of the Clinical and Laboratory Standards Institute and the International Federation of Clinical Chemistry [38].

This study was undertaken in a well-defined patient population seen and evaluated in an ambulatory bariatric clinic at our academic institution and is in agreement with similar prior studies in terms of the high prevalence of hepatic steatosis and NASH, and the low prevalence of advanced fibrosis [13,29,30,31,32,33,34,35,36] in this patient population. The main strengths of this study lie in its prospectively collected high-quality data, the fact that liver biopsies were performed intraoperatively at the time of the metabolic surgery, and blood samples were collected under the same circumstances in the same day of surgery. Further, all blood chemistry analyses were performed in the same clinical laboratory, thus avoiding the potential differences in reporting the results. Finally, all liver biopsies were interpreted by an experienced liver pathologist who was blinded to the clinical and laboratory backgrounds of the participating patients. Our study has some limitations, as is typical of populations of individuals undergoing metabolic surgery, the majority of patients were women [13]. Further, most of the participants were of Caucasian race which mirrors the racial make-up of the Midwest region of the U.S. In addition, the prevalence of the significant liver fibrosis group was small likely due to the nature of the study in this patient group who presents for elective bariatric surgery as all subjects were recruited from a bariatric clinic. Neither full liver screening tests at the time of surgery to rule out other etiologies that cause elevated liver enzymes nor fractionation of serum ALP were performed because the recruited subjects fit the profile of those with NAFLD/NASH, and other causes of liver disease were excluded based on history, existing laboratory data in patients records, and histological evaluation by an experienced pathologist. Therefore, additional screening testing prior to surgery was felt to be unnecessary. We emphasize that the interpreting pathologist assessed all liver biopsy specimens for the etiologies commonly leading to abnormal liver chemistry. Finally, our results will require external validation in larger cohorts.

## 5. Conclusions

Serum ALP, in addition to ALT, HbA1c, and BMI, was found to be an independent predictor of significant fibrosis in obese subjects with NAFLD. We introduce a new tree-based model using the four noninvasive significant variables for prediction of significant liver fibrosis, which, if validated, may be used for patient counseling and for identifying high-risk patients who might benefit from intraoperative liver biopsy for staging in patients undergoing metabolic surgery.

## Figures and Tables

**Table 1:** Baseline clinical and laboratory features of the study participants.

Variable	All Subjects (*n* = 210)	F0–F1 (*n* = 189)	F2–F4 (*n* = 21)	*p* Value
Age, years				
Median	46	45	53	0.09
(Range)	(22.9–77.2)	(22.9–77.2)	(25–65.7)	
Gender, female				
Percentage	82.9%	82%	90.5%	0.33
Frequency	174	155	19	
Tobacco smoking, yes				
Percentage	46.6%	46.5%	47.6%	0.92
Frequency	97	87	10	
Body weight, kg				
Median	129.1	129.1	138	0.39
(Range)	(87.2–238.9)	(87.2–238.9)	(106.9–173)	
Body mass index, kg/m^2^				
Median	46.2	46	52.7	0.19
(Range)	(33.2–67.3)	(33.2–67.3)	(38–63)	
Diabetes mellitus *, yes				
Percentage	32.7%	28.9%	66.7%	<0.0001
Frequency	68	54	14	
Hypertension ^†^, yes				
Percentage	54.3%	52.4%	71.4%	0.1
Frequency	113	98	15	
Hyperlipidemia ^‡^, yes				
Percentage	43.8%	41.7%	61.9%	0.08
Frequency	91	78	13	
Glucose, mg/dL				
Median	94	92	120	0.0002
(Range)	(57–283)	(57–238)	(75–225)	
HbA1c, %				
Median	5.8	5.7	7.3	<0.0001
Range	(4.3–13.2)	(4.3–13.2)	(5.2–11.8)	
Albumin, g/dL				
Median	4.3	4.3	4.6	0.08
(Range)	(3.4–5.4)	(3.4–5.3)	(4–5.4)	
ALP, U/L				
Median	69	67	87	0.0015
(Range)	(26–157)	(26–157)	(53–127)	
AST, U/L				
Median	26	25	38	0.0007
(Range)	(9–152)	(9–152)	(17–125)	
ALT, U/L				
Median	29	27	43	0.0015
(Range)	(9–273)	(9–186)	(14–273)	
AST/ALT ratio				
Median	0.89	0.89	0.88	0.46
(Range)	(0.35–1.92)	(0.35–1.92)	(0.46–1.64)	
Hb g/dL				
Median	13.6	13.5	14.2	0.046
(Range)	(9.5–16.7)	(9.5–16.4)	(12–16.7)	
Platelets, cell × 10^9^				
Median	268	268	283	0.79
(Range)	(88–510)	(88–510)	(117–437)	
TC, mg/dL				
Median	160	159	172	0.62
(Range)	(77–294)	(104-294)	(77–244)	
TG, mg/dL				
Median	123	120	139	0.048
(Range)	(37–454)	(37–454)	(78–243)	
LDL, mg/dL				
Median	97	96	106	0.99
(Range)	(21–231)	(35–231)	(21–167)	
HDL, mg/dL				
Median	39	39	37	0.71
(Range)	(20–82)	(20–82)	(27–64)	
LDL/HDL ratio				
Median	2.48	2.56	2.13	0.17
(Range)	(0.6–8)	(0.6–8)	(0.6–6.2)	
TC/HDL ratio				
Median	4.16	4.2	3.68	0.54
(Range)	(1.2–10.1)	(1.2–10.1)	(2.8–8.3)	

* Data available for 208 patients. ^†^ Data available for 208 patients. ^‡^ Data available for 208 patients. Abbreviations: ALP, alkaline phosphatase; AST, aspartate aminotransferase; ALT, alanine aminotransferase; Hb, hemoglobin; TC, total cholesterol; TG, triglycerides; LDL, low-density lipoprotein; and HDL, high-density lipoprotein.

**Table 2:** Baseline liver histological features of the study participants.

Biopsy Finding	% (Frequency)
Steatosis	
<5%	13% (27)
5–33%	27% (58)
66%	40% (83)
>66%	20% (42)
Lobular inflammation *	
None	43% (92)
<2 foci	46% (97)
2–4 foci	10% (20)
>4 foci	1% (1)
Ballooning	
None	61% (129)
Few	31% (64)
Many	8% (17)
NAS	
0	14% (29)
1	18% (37)
2	11% (24)
3	19% (41)
4	16% (33)
5	15% (32)
6	5% (12)
7	2% (2)
Fibrosis stage	
0	71% (150)
1	19% (39)
2	4% (8)
3	5% (10)
4	1% (3)

* Lobular inflammation was determined per 200× filed. Data are from *n* = 210 patients. Abbreviations: NAS, nonalcoholic fatty liver disease activity score.

**Table 3:** Parameter Estimates for a multivariable assessment of fibrosis risk factors.

Parameter	Estimate	Odds Ratio		
Intercept	−11.86	**Estimate**	**95% Confidence Interval**	
HbA1c	0.477	1.61	1.17	2.09
BMI	0.066	1.07	1.00	1.16
ALP	0.031	1.03	1.01	1.06
ALT	0.021	1.02	1.01	1.05

Data are from *n* = 210 patients. Abbreviations: BMI, body mass index; ALP, alkaline phosphatase; ALT, alanine aminotransferase.

## Data Availability

The data presented in this study are available on request from the corresponding author. The data are not publicly available to protect the privacy of the participating subjects as outlined in our study protocol.
